# Presence of Potentially Toxic Elements in Historical Mining Areas in the North-Center of Mexico and Possible Bioremediation Strategies

**DOI:** 10.3390/toxics12110813

**Published:** 2024-11-13

**Authors:** Victor Manuel Escot-Espinoza, Susana Rodríguez-Márquez, Jorge Briseño-Bugarín, Maria Argelia López-Luna, Juan Armando Flores de la Torre

**Affiliations:** 1 Toxicology and Pharmacy Laboratory, Health Sciences Area, Academic Unit of Chemical Sciences, Autonomous University of Zacatecas, Zacatecas 98160, Mexico; victor.escot@uaz.edu.mx (V.M.E.-E.); jbriseno@uaz.edu.mx (J.B.-B.); mariaa.lopez@uaz.edu.mx (M.A.L.-L.); 2 Secretary of Water and Environment of the State of Zacatecas, Building F, Cerro del Gato Circuit, Administrative City, Zacatecas 99160, Mexico; sama@zacatecas.gob.mx

**Keywords:** potentially toxic elements, mine wastes, environmental receptors, bioremediation

## Abstract

This paper provides an overview of the impacts of mining-related environmental liabilities on humans, soils, sediments, surface water and groundwater across various mining districts in Zacatecas, Mexico. An analysis has been carried out on the areas of the state most affected by the presence of potentially toxic elements (PTEs) such as arsenic, lead, cadmium, copper, chromium and zinc, identifying priority areas for environmental assessment and remediation. Likewise, a review of the concentrations of PTEs reported in different environmental matrices of the state’s mining areas with the presence of environmental liabilities was carried out, most of which exceed the maximum permissible limits established by Mexican and international regulations, generating an environmental risk for the populations near these districts due to their potential incorporation into the food chain. Additionally, this study explores research focused on the biostabilization of PTEs using microorganisms with specific metabolic activities. Phytoremediation is presented as a viable tool for the stabilization and elimination of PTEs, in which endemic plants from arid–semi-arid climates have shown favorable results in terms of the phytostabilization and phytoextraction processes of the PTEs present in mining waste.

## 1. Introduction

Mining in Mexico dates to pre-Hispanic times, acquiring great economic and social relevance during the colonial period, producing materials for different industries and generating infrastructure in mining regions in the north of the country [1]. However, over nearly 450 years of activity, this industry has generated millions of tons of solid waste, primarily from mining and metallurgical processes, such as amalgamation and cyanidation for the recovery of gold (Au) and silver (Ag). This has resulted in deposits of mining waste in dams or mounds containing high concentrations of potentially toxic elements (PTEs). These are exposed to environmental conditions and their condition and potential effects are unknown [2].

There are 12 mineralized regions in Mexico, and more than 76% of its states carry out some type of mining activity. Mexico is the largest producer of Ag worldwide and ranks among the top 10 producers of minerals such as gold (Au), lead (Pb), zinc (Zn), cadmium (Cd) and copper (Cu), among others [3]. One of the most important mining areas in Mexico is the state of Zacatecas, where it is currently the main economic activity [4]. This state is divided into 17 mining regions according to the type of mineralization and geographic location (Table 1). Metallogenic events that form mining districts are present in these areas, among which the following stand out: Concepción del Oro, Mazapil, Fresnillo, Miguel Auza, Ojocaliente, Sombrerete and Zacatecas mining districts [3,5].

Due to the mining history of the state of Zacatecas, large amounts of mining waste have been generated in the mining districts; however, there is a lack of information regarding its geochemical–environmental characteristics. In 2017, the Information System for Contaminated Sites (SISCO, by its acronym in Spanish) reported the presence of 112 mining waste sites as potential environmental liabilities in the state [6]. This type of waste site is characterized by a lack of prevention and pollution control programs throughout its operation, as well as an absence of restoration measures, resulting in an environmental problem that affects the population, especially considering demographic growth and the proximity of the communities to this waste.

This review focuses on the existing information regarding historical mining waste sites present in the state of Zacatecas, their characteristics and concentrations of PTEs, and their effects on the different environmental receptors. It also provides a general overview of the bioremediation strategies that have been evaluated at sites contaminated by mining–metallurgical activities in the state.

### Search Strategy and Data Collection

To narrow the focus of this work, the following research questions are formulated: “What concentrations of PTEs have been determined in historical mining areas of the state of Zacatecas?” and “what bioremediation strategies have been implemented to mitigate the adverse effects of the presence of PTEs?” Therefore, our review focuses on quantification studies of PTEs in different environmental matrices of mining areas of the state of Zacatecas and the main bioremediation strategies. The inclusion criteria were (1) reports from mining companies, (2) original research articles, (3) articles published until august 2024 and (4) works written in English or Spanish. The search strategy consisted of using keywords such as Zacatecas, Mexico, potentially toxic elements, mining waste and bioremediation; Boolean operators, such as “potentially toxic elements OR PTEs AND mining waste AND Mexico AND Zacatecas,” were also used in the research databases Web of Science, Scopus, PubMed, Google Academic and ScienceDirect. The results were classified based on the information to be incorporated into the following sections: Section 2, regarding environmental implications due to the presence of mining passives; Section 3, regarding the impact of PTEs in mining districts of Zacatecas or bioremediation proposals at sites impacted by mining waste in Zacatecas; and Section 4, regarding bioremediation proposals at sites impacted by mining waste in Zacatecas.

## 2. Environmental Implications Due to the Presence of Mining Passives

### 2.1. Environmental Assessment of Mining Passives

PTEs include all those elements that, due to their characteristics and concentrations, can be toxic to biological systems. The evaluation of antimony (Sb), arsenic (As), beryllium (Be), Cd, chromium (Cr), Cu, mercury (Hg), nickel (Ni), silver (Ag), Pb, selenium (Se), thallium (Tl) and Zn is a priority in Mexico [7]. These PTEs can be present in the form of metal cations, native metals, oxyanions, halides and organo-complexes, making their environmental assessment in heterogeneous systems, such as those of mining wastes, difficult [8].

Mining waste can be divided into two categories: (i) waste rock produced during exploration and discovery of the deposit and (ii) mining waste generated during the processing of the mineral. Many mineral processing methods involve grinding rocks and ores, recovering the desired fraction and removing and depositing the waste in tailings dams or mounds, and over 99% of the original material can become waste when processing low-grade ores [9,10]. Typically, tailings dams are built aboveground for the storage of mine waste, but in arid and semi-arid regions, wetting the surface to prevent wind erosion is not practical, especially after the closure of mining operations; hence, historical mining waste is a source of pollution in the form of particulate matter, which is measured in fractions of ≤10 μm (PM_10_) and ≤2.5 μm (PM_2.5_) aerodynamic diameter [11]. Short-term exposure to PM_10_ and PM_2.5_ particles in the air causes premature death in people with heart or lung disease, other respiratory conditions and decreased lung function, while long-term exposure can cause lung cancer and chronic respiratory diseases [12].

Understanding the interactions between waste and water, as well as the mineralogical characterization of the materials, is needed for more accurate prediction of the environmental impact of mining waste. A first step in understanding the geochemical behavior of hazardous wastes is to determine their acidity production and neutralization potential, which, in Mexican regulations, are together referred to as net neutralization potential and indicate the capacity of waste to generate acid mine drainage (AMD) [7,10]. AMD is generated through the oxidation of sulfide-type minerals, which, in the presence of meteoric water, generates a low-pH solution that promotes the dissolution of other mineralogical phases containing PTEs. This process disperses particles or elements in solution into abiotic environments, primarily soil, surface water and groundwater [13].

In addition, PTEs present in mining wastes can be mobilized by chemical release mechanisms (dissolution and/or leaching) or by physical transport (by wind and/or water). These processes are favored in arid–semi-arid areas as a consequence of rain–drought cycles and physicochemical conditions such as pH, redox potential, humidity and organic matter content [14,15,16].

### 2.2. PTEs in Areas with Historical Mining Waste Worldwide

Globally, due to the various environmental liabilities left by abandoned mining areas and the exploitation of mineral resources, large areas of soil have been contaminated with PTEs [17]. The ecological risks of PTE contamination in soil are significant, and various authors have carried out work in which they show the large number of impacted areas at both regional and global levels [18,19,20]. At present, the mining and smelting of metallic minerals, as well as the abandonment of mining sites, are the main contributors to soil, water and sediment contamination by PTEs [21]. Table 2 presents some historical mining sites with high PTE contents.

Historically, mining has served as a viable route to national development in most resource-rich countries, such as Australia, China and the United States of America, where mining has been the main driver of economic growth and industrialization; however, countries such as Mexico, Spain, Italy, France and some countries in Africa also experience contamination due to the presence of mining environmental liabilities with high PTE contents [17]. Table 2 shows the presence of different contaminated environmental receptors, where concentrations have been determined in soils close to mining areas of up to 4771 mg/kg of As in Turkey [35], 14,500 mg/kg of Pb in Tunisia [34], 486 mg/kg of Cr in Oman [39], 225 mg/kg of Cd in France [29], 8980 mg/kg of Cu in Zambia [40], 32,287 mg/kg of Zn in Spain [32] and up to 170 mg/kg of Hg in the USA [23]. High concentrations of Pb, Cd and Zn have been found in river sediments, mainly in mining areas of Spain, Australia and Mexico [43,44,45,46]. Finally, water samples impacted by mining waste have been primarily evaluated in the United States, where contamination associated with the presence of As has been observed [47].

### 2.3. Overview of PTE Pollution in the State of Zacatecas, Mexico

It has been estimated that 94% of the waste generated by the mining–metallurgical industry in Mexico is concentrated in the states of Sonora, Zacatecas, Chihuahua, Durango, San Luis Potosí, Querétaro and Coahuila [6]. In this regard, technical standards have been issued for the control of environmental impacts, with NOM-141-SEMARNAT-2003 [7] being the first official Mexican standard to be established. This standard applies only to mining waste generated in projects from 2003 onwards, requiring the company to carry out characterization studies of the final disposal site. Therefore, the environmental problems caused by the mining industry in previous years are still present at various historical mining waste sites.

**Figure 1:**
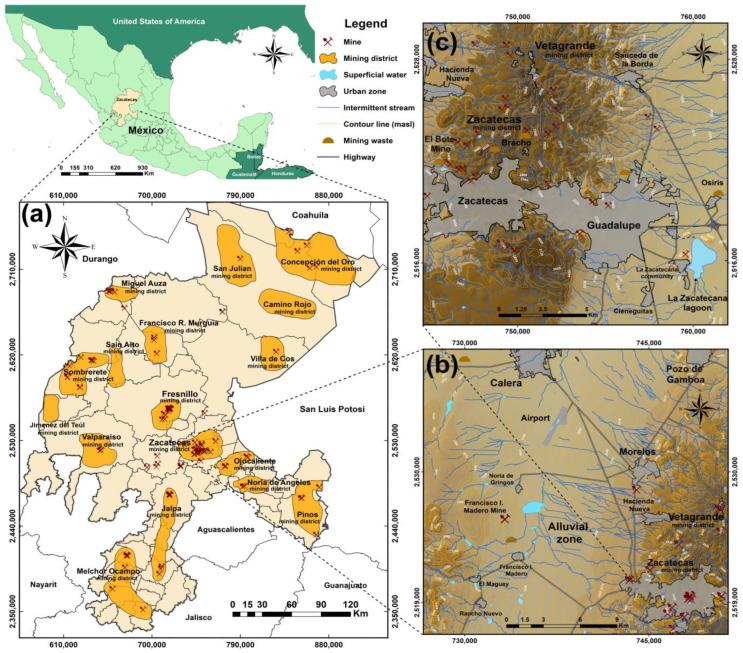
Map of the study area showing (**a**) the main mining districts of the state of Zacatecas, (**b**) the sediment accumulation zone in the Calera-Francisco I. Madero-Zacatecas valley and (**c**) the area of the Zacatecas and Vetagrande mining districts and La Zacatecana lagoon.

The state of Zacatecas has a territorial area of 77,684 km^2^ and is divided into 58 municipalities (Figure 1a). Its predominant climate (60% of its territory) is of the Bs type, or dry–semi-arid, which is characterized by scarce rainfall, moderate temperatures in summer and low temperatures in winter. The northeast of the state (~24% of its territory) primarily has a Bw or dry–arid climate [48]. The soil contamination in the semi-arid region of the state has its origin in the ancient mining–metallurgical activities involved in the recovery of Ag that were carried out through the “Patio Process” between 1550 and 1900 and which consisted in the formation of a cold silver amalgam using Hg (quicksilver). This recovery process was used intensively in the state, and the levels of Hg contamination associated with the waste generated from the process are unknown; limited studies have reported concentrations of up to 169 mg/kg of Hg in soils in the state [49].

The Zacatecas mining district was discovered in 1546, and it has been estimated that its Ag production from 1548 to 1987 was approximately 23,236,499 kg; during this period, grades of up to 486 g/t of Ag, 4.8 g/t of Au, 26,400 g/t of Pb and 62,800 g/t of Zn were determined [50]. Table 3 shows an estimate of the mining waste generated in the Zacatecas mining district from metal ore mining processes between 1982 and 2014, amounting to ~17,025,059,486 tons of rock waste. This material is possibly distributed in the vicinity of the cities of Zacatecas and Guadalupe, with uncertainty regarding how much of this material was properly treated [51,52].

The semi-arid climate that prevails in the state could favor the hydric and aeolian dispersion of PTE-carrying particles. It is known that the main erosive agent affecting the cities of Zacatecas and Guadalupe is rainwater, which has generated zones of sediment accumulation due to fluvial effects in the El Bote mine, the communities of Bracho, Vetagrande, Panuco, Francisco I. Madero, Noria de Gringos and the city of Zacatecas [53]. These areas are characterized by the presence of mining passives, unconsolidated sediments, fluvial deposits and alluvial valleys (Figure 1b).

To better understand the environmental implications of the presence of mining waste in the state of Zacatecas, thematic maps were made using geostatistics, in conjunction with geographic information systems (GIS), which facilitate the assessment of the level of contamination in soils and sediments, the identification of pollutant dispersion patterns and the identification of areas of interest and priority.

**Figure 2:**
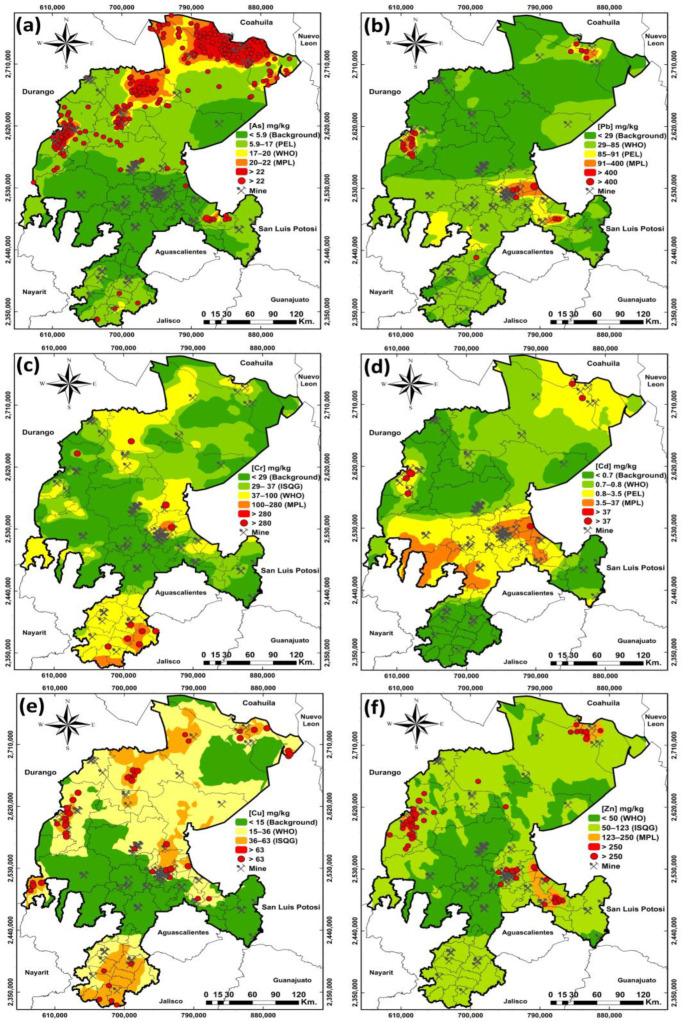
Isoconcentration maps generated for (**a**) As, (**b**) Pb, (**c**) Cr, (**d**) Cd, (**e**) Cu and (**f**) Zn using the Kriging interpolation method. The degree of contamination is presented based on the MPL established in Mexico by the Federal Official Gazette (known by its Spanish abbreviation—DOF) and the World Health Organization (WHO) [56,57], as well as the probable effect level (PEL) and the interim sediment quality guideline (ISQG) established by the Canadian Council of Miners of the Environment (CEQG) [55].

The maps in Figure 2 were generated using ArcMap 10.3.1 software, utilizing PTE concentrations reported in the geochemical charts provided by the Mexican Geological Survey [54]. To create isoconcentration maps, sample values for the elements As, Pb, Cr, Cd, Cu and Zn (*n* = 2104) were treated and analyzed using the ordinary Kriging interpolation geostatistical method. The number of intervals in the map was generated based on the maximum permissible limits (MPL) established in the Mexican regulations for soil and the international regulations for sediments [55,56].

Figure 2 shows that the mining districts of the state most affected by the presence of PTEs are the Zacatecas and Vetagrande mining districts, located in the center of the state, with the presence of concentrations of Pb, Cr, Cd, Cu and Zn above the MPL established by national and international regulations. Towards the north of the state, the Mazapil and Concepcion del Oro mining districts show samples above the MPL for As, Pb, Cd, Cu and Zn, with a considerable number of samples presenting high concentrations of As in these districts, together with the Miguel Auza, Francisco R. Murguía and Sombrerete mining districts. Specifically, the Sombrerete mining district presents high concentrations of As, Pb, Cd, Cu and Zn. Finally, in the southern part of the state, the mining district most affected by the presence of As, Pb, Cd and Zn is the Noria de Angeles mining district, while the mining district most affected by the presence of Cr is Mezquital del Oro. These affected districts were evaluated in the papers presented in this review.

This methodology was also used to evaluate the dispersion of Pb in soils of the Vetagrandre mining district, where soils from impacted residential areas such as kindergartens, recreational areas and the downtown area have been identified with concentrations of 8 to 7672 mg/kg [58].

## 3. Impact of PTEs in Mining Districts of Zacatecas

### 3.1. Contamination by PTEs in Aquifers in the State of Zacatecas

Two problems in the arid and semi-arid zones of northern Mexico are water shortage and overexploitation of aquifers. Although it is known that agriculture is the main source of water consumption in the state of Zacatecas, some studies have focused on evaluating the relationships that mining districts have to the recharge deficits of aquifers. Guzmán-López [52] carried out a balance of the 34 available aquifers in the state of Zacatecas, which together receive a recharge of 1026 million m^3^ of water per year. The study observed that 14 aquifers show a deficit or overexploitation close to 435 million of m^3^, equivalent to 29.2% of the total annual recharge of the 34 aquifers. The study highlights that municipalities with mining districts, such as Fresnillo, Noria de Angeles, Vetagrande, Concepcion del Oro, Mazapil and Sombrerete (Figure 1a), report deficits in water availability.

Leaching and infiltration of water with contaminant-bearing particles has had a negative impact on the quality of groundwater, surface water, shallow water wells and the soil quality of the ecosystems surrounding the mines [46]. Water pollution in places such as the Zacatecas–Guadalupe suburban zone is related to the presence of heavy metals and other elements such as As and fluoride (F^−^). NOM-127-SSA1-1994 establishes an MPL of 1.5 mg/L for F^−^ and 0.025 mg/L for As in drinking water [59]. Davila [60] has evaluated the presence of As and F^−^ in the three water supply systems in the suburban area of Zacatecas–Guadalupe and found an average concentration of 71 ± 117 µg/L of As in well and drinking water samples, which is 16 times above the MPL established by Mexican regulations and 40 times above the MLP established by the WHO (10 µg/L) [57]. They also determined an average F^−^ concentration of 1.58 ± 0.63 mg/L, with samples exceeding up to twice the MPL established by the Mexican regulations (Table 4).

Studies in the south-central area of the state of Zacatecas have shown the risk to children in public schools when consuming water with high contents of As and F^−^. In the Ojocaliente area, the maximum As concentrations detected in public school water were 298 µg/L, and maximum concentrations of F^−^ of 3 mg/L in Tabasco, 2.4 mg/L in the community of El Visitador and 2.3 mg/L in the municipality of Jerez were observed (Table 4) [61].

Another area in the state that has encountered issues due to the presence of As and F^−^ is the plain between the municipalities of Zacatecas, Calera and Morelos, which is located near the Zacatecas formation (Figure 1b). Navarro et al. [62] evaluated the presence of As and F^−^ in samples of groundwater used for human consumption and determined that 68% of the samples exceeded the MPL established by the WHO for As, attributed to both an anthropic origin, associated with mining activities, and a natural origin, associated with geological structures of the quaternary alluvium type. The highest concentrations of As were detected in the Maderos mine, the El Bote mine and the alluvial zone of Calera (between 23.1 and 75.4 µg/L of As). Nearly 22% of the samples exceeded the MPL for F^−^, which was attributed to the presence of fluorite (CaF_2_). Values above the permissible limit for As and F^−^ were also detected in the south of the study area, where there is intense agricultural activity and the use of phosphate pesticides is a common practice. Although there are anthropogenic sources, most of the groundwater contamination is of geological origin, with As associated with oxidation reactions in sulfide minerals found in metasedimentary rocks, while F^−^ results from water–rock interactions with CaF_2_ (Table 4).

In this same alluvial zone (Figure 1b), the presence of As, Pb and Hg in agricultural irrigation waters has been evaluated, and average concentrations for As, Pb and Hg of 158 ± 49 µg/L, 353 ± 21 µg/L and 2.7 ± 0.4 µg/L, respectively, have been determined (Table 4) [63]. This area has mining waste sediments dispersed in the vicinity of the Francisco I. Madero mine, which form an alluvial fan of 5 km^2^ and currently affect crop areas and surface water bodies; however, the origin of these sediments has not yet been determined.

Another aquifer that has been contaminated by PTEs is the Guadalupe–Bañuelos aquifer, to which the La Zacatecana lagoon and the city of Guadalupe belong. In this area, Padilla-Reyes et al. [66] evaluated the groundwater quality and classified it as potable, as the pH values, total dissolved solids, chlorides, sulfates, Pb and Hg are below those established by the Mexican regulations; however, they present samples with As and F^−^ values higher than the MPL (Table 4). In their study, it was determined that the rainfall flows that were evaluated converge in La Zacatecana lagoon, which is a historical mining site widely studied for the presence of high concentrations of PTEs in soil and sediments; this site is further described in Section 3.2.

### 3.2. PTE Contamination in Soils of Zacatecas Mining Districts

Although the dispersion of PTEs from mining wastes can severely affect all environmental receptors, soil pollution is of particular importance, as it acts as a natural regulator of the transport of elements and chemical substances into the atmosphere, hydrosphere and biosphere [68]. Mine waste can contain significant amounts of PTEs; however, the portion of the total content of a PTE released from the solid phase through mechanical, chemical or biological processes must be considered as the geo-available portion, which is governed by abundance, exposure to weathering factors and the susceptibility of the carrier mineral phases to be modified geochemically by the effect of pH, oxidation reduction potential, sorption reactions, solubility reactions and AMD generation, or biologically through biotransformation and bioaccumulation processes, which favor the redistribution of the PTEs in different environmental receptors [69].

The Fresnillo mining district is one of the most important zones in Zacatecas (Figure 1a). There, mining has developed for more than 450 years, standing out as the main producer of Ag worldwide, partly due to the application of the patio beneficiation method, cyanide leaching and, currently, the flotation process (Figure 1a). Although the amount of waste generated by each type of process throughout the site’s history is unknown, a considerable amount of waste has been dispersed in soils in residential areas of the city of Fresnillo and nearby communities. At these sites, concentrations of up to 1219 mg/kg of As, 6100 mg/kg of Pb and 292 mg/kg of Hg have been determined. Likewise, the concentration of PTEs in Fresnillo mining district sediments has been evaluated, presenting concentrations of As up to 152 mg/kg, Pb up to 345 mg/kg and Hg up to 20 mg/kg; surface water bodies show concentrations of up to 380 µg/L of As, 84 µg/L of Pb and 50 µg/L of Hg (Table 5) [64]. The deposits are mainly composed of metallic sulfides of sphalerite (ZnS), galena (PbS), chalcopyrite (CuFeS_2_), bornite (Cu_5_FeS_4_), argentite (Ag_2_S), polybasite (Ag_3_SbS_3_), pyrrhotite (Fe_1−xS_), pyrite (FeS_2_) and arsenopyrite (FeAsS) [70], which implies a latent risk due to the possible formation of AMD in the deposition zone of these mining wastes.

On the other hand, one of the most evaluated areas in the Zacatecas mining district is the El Bote mine (Figure 1c), which has generated mining waste dating back approximately 300 years, affecting an area of 1470 m^2^ and being part of the urban area of the city of Zacatecas [71]. During its first period of exploitation (1912–1943), the Ag and Au amalgamation process was used. Subsequently, in the period between 1972 and 1993, a flotation concentrator plant was established for the beneficiation of Pb, Zn and Cu in an area rich in metallic sulfides of ZnS, PbS, CuFeS_2_, Ag_2_S, Ag_3_SbS_3_ and FeS_2_ [3]. It is estimated that, between 1912 and 1943, 1,317,600 tons of oxidized ores were extracted with grades of 100 g/t Ag and 1.1 g/t Au [72]. It has been reported that, in 1956, the tailings dam of the El Bote mine had a spill, affecting agricultural lands in the surrounding communities due to the fluvial transport of particulate matter [73]. In the area, average concentrations of 2621 ± 53 mg/kg of Pb and 3827 ± 83 mg/kg of Zn have been determined, with maximum concentrations of 8466 ± 116 mg/kg of Pb and 12,475 ± 324 mg/kg of Zn (Table 5). Based on the geo-accumulation index, the area has been classified as highly contaminated for Pb and Zn, with rhizospheric soil classified as moderately contaminated, which has generated a beneficial effect on plants present in the tailings (*Cortaderia selloana* and *Sporobolus airoides*) [71].

It is known that considerable amounts of the PTEs present in the historic mining sites of the Zacatecas mining district where the amalgamation process was used (1570 to 1820) were transported by intermittent river flows and deposited in the flat areas of the Zacatecas–Guadalupe valley (Figure 1c) [74,75].

La Zacatecana is a community in the municipality of Guadalupe (Figure 1c), where several authors have reported the presence of alluvial mining residues in agricultural and livestock soils. Soil concentrations of 47.9 to 868 mg/kg of Hg related to the Au and Ag amalgamation process have been determined in the area. The Hg is present in fractions related to elemental-amalgamated mercury, Fe and Mn oxides and sulfides (HgS) [76]. The potential risk in La Zacatecana lagoon is estimated to be low, due to the high stability and low mobility of its Hg species; however, Hg is not the only PTE that has been found in the area. Covarrubias et al. [77] evaluated the mobility of heavy metals present in soils and sediments of this lagoon, identifying a sequence from higher to lower mobility of Pb > Cr > As > Ni > Hg > Cd. They determined concentrations of 3070 ± 20 mg/kg of Pb, 67 ± 3.6 mg/kg of Cr, 101 ± 2.8 mg/kg of As, 47 ± 11 mg/kg of Hg and 21 ± 0.1 mg/kg of Cd. Under conditions of high concentrations of Pb, it presents motilities of up to 29%, associated with exchangeable fractions and carbonates (Table 5). The calculated geo-accumulation index values suggest that the La Zacatecana dam has experienced high contamination by Pb and moderate to strong contamination by As [77]. It has been established that the origin of the high concentrations of PTEs in La Zacatecana lagoon are associated with the mining activities developed since 1920, with biomagnification promoted by the intermittent rivers coming from the Sierra de Zacatecas and the flow of the Arroyo de la Plata into the lagoon basin, as a dispersion route for PTEs coming from the Zacatecas and Vetagrande mining districts (Figure 1c) [76,78].

Another site of environmental interest in the state of Zacatecas is the Concepcion del Oro mining district, where skarn polymetallic deposits have been exploited for more than 400 years, with the presence of residues from hydrometallurgical and pyrometallurgical processes of Au, Ag, Cu, Pb and Zn, obtained from an area rich in CuFeS_2_ and FeS_2_. Five mining waste deposits and one slag deposit have been identified at the site near the urban area of the community of Concepción del Oro, covering an area of 0.5 km^2^. Concentrations of up to 500 mg/kg of As and 600 mg/kg of Pb were determined in soil and stream sediments, suggesting that contaminated sediments are dispersed during the dry season to the Arroyo Principal, which represents the only source of water for the agricultural activities of the community [15,79].

### 3.3. Studies of PTEs Incorporated into Food Chains

Different biological markers, including venous and capillary blood, umbilical cord blood, plasma, urine, teeth, bones and hair, can be used to determine the level of exposure to PTEs. In this regard, González-Valdez et al. [67] determined the concentration of Pb in the blood of 80 children in the Vetagrande mining district; 55% of the samples had concentrations that represent a serious health risk (>10 ug/dL). They mention that the presence of Pb in blood is because the houses of that population are located on land enriched with Pb.

On the other hand, as plant absorption is one of the main pathways through which PTEs enter the food chain, it is essential to monitor the quality of food for human and animal consumption [80].

In Zacatecas, it is common to observe agricultural and livestock areas near mining districts, so it is important to determine the impact on crop soils, grazing areas and water supply sources. The dispersion of Pb to agricultural soils near a Pb recycling plant in the community of San Ignacio in the municipality of Fresnillo has been evaluated. The plant used the bismuth recovery process, and Pb concentrations have been determined in the source of up to 84,238 mg/kg; in agricultural and residential soils of 4940 ± 14,950 mg/kg, in plants for human consumption, such as oats, nopal and chili of 16,220 ± 20,954 mg/kg; in medicinal plants of 530 ± 30 mg/kg; and in wild plants (*Buddleja*, *Acacia*, *Prosopis* and *Opuntia*) of up to 39,926 mg/kg (Table 5) [81].

Salas-Muñoz et al. [82] determined concentrations of As up to 165 ± 7.4 mg/kg and Pb up to 1206 ± 155 mg/kg in agricultural soils where vegetables (carrot, bell pepper and garlic) are grown; these concentrations exceed the standards established in national and international health and food safety regulations (Table 5). The evaluated cultivation areas are in communities with historical mining waste near the city of Zacatecas.

**Table 5:** PTE concentrations determined in soil and sediment samples in the mining areas of the state of Zacatecas.

Location	Type of Sample	Sample Processing and Analytical Technique	Concentration (mg/kg)								Ref.
			As	Pb	Hg	Cr	Cd	Cu	Zn	Mn	
Santa Rita	Agricultural soils adjacent to areas with the presence of mining waste	Microwave-digested samples. Analyzed using FAAS ^(2)^	135 ± 20	179 ± 27.3							[82]
El Bordo			141 ± 19.2	1201 ± 130							
El Lampotal			138 ± 8.43	185 ± 13.5							
La Era			165 ± 7.35	1206 ± 155							
Maderos’s mine	Waste with presence of vegetation and fungi	Microwave-digested samples. Analyzed using FAAS ^(2)^		67 to 120							[83]
El Bote mine				73 to 96							
Vetagrande				49 to 163							
Old Jal				Until 143							
San Martin-Sombrerete mining district	Composed of mining waste	Hot-plate-digested samples. Analyzed using FAAS ^(2)^	2004	132							[84]
	Flotation tailings		1101	113							
Noria de Ángeles mining district	Red mining waste	Microwave-digested samples. Analyzed using ICP-OES ^(1)^	5199 ± 3	820 ± 137				476 ± 4	2454 ± 373	493 ± 74	[85]
	Grey mining waste		3958 ± 286	1239 ± 103				136 ± 11	1284 ± 234	756 ± 16	
La Zacatecana lagoon	Soils and sediments	Hot-plate-digested samples. Analyzed using FAAS ^(2)^	101 ± 2.8	3070 ± 20	47 ± 11	74 ± 2.5	21 ± 0.1				[77]
Noria de Ángeles mining district	Soil	Hot-plate-digested samples. Analyzed using FAAS ^(2)^		50 to 429			1 to 4	30 to 95	60 to 250		[86]
	Mine waste			~2000			~20	~450	~1000		
El Bote mine	Mine waste	Hot-plate-digested samples. Analyzed using FAAS ^(2)^		Ave: 2621 ± 53 Max: 8466 ± 116					Ave: 3827 ± 83 Max:12,475 ± 324		[71]
Fresnillo mining district	Recreational park waste	Microwave-digested samples. Analyzed using FAAS ^(2)^	882	477							[87]
	Recreational park plant sprouts		Ave: 499 Max: 1050	Ave: 7.2 Max: 16							
Community of San Ignacio, Fresnillo	Processing plant	Samples dried between 40 and 60 °C. Analyzed using EDXRF ^(4)^		84,238							[81]
	Agricultural–urban soils			4940 ± 1950							
	Plants for consumption			16,220 ± 20,954							
	Medicinal plants			530 ± 30							
	Wild plants			368 to 39,926							
	Mud vessel			30,443 ± 1808							
El Bote Mine	Soils used as substrate to evaluate germination and biomass growth of plants	Hot-plate-digested samples. Analyzed using ICP-OES ^(1)^	0.67	3.89	0.05						[88]
Noria de Ángeles			7.42	6.17	0.10						
Sombrerete			26.2	69.67	0.08						
Vetagrande			1.65	20.81	0.05						
El Bordo			1.73	5.35	0.05						
Vetagrande mining district	Composting of waste (phytostabilization)	Microwave-digested samples. Analyzed using FAAS ^(2)^		3518 ± 199		69.5 ± 5	56.2 ± 2	220 ± 12	7674 ± 292		[89]
	Old waste		305 ± 18	3836 ± 73	2464 ± 225	17.7 ± 3	73.6 ± 2	329 ± 5	8746 ± 194		
Fresnillo mining district	Soils near mining waste	Microwave-digested samples. Analyzed using FAAS ^(2)^ and HGAAS ^(3)^	2 to 1219	4 to 6100	0.1 to 292		0.1 to 47	6 to 1965	12 to 5341	75 to 3411	[64]
	Sediments		7 to 152	23 to 345	0.02 to 20		0.2	22 to 77	105 to 426	50 to 271	
Community of Vetagrande	Kindergarten soils	Samples dried to 40 °C. Analyzed using EDXRF ^(4)^		1901							[58]
	Recreational areas			1489							
	Downtown area			724							
Concepción del Oro	Mining waste	Samples digested with boric acid and sodium metaborate. Analyzed using ICP-OES ^(1)^	70 to 515	16.4 to 200				27.7 to 1348	87.7 to 347		[15]
Francisco I. Madero	Soil	Samples dried to 60 °C. Analyzed using EDXRF ^(4)^		699 to 2898							[5]
	Shoot of *Amaranthus hybridus*			2208 ± 136							
	Shoot of *Buddleja scordioides*			1378 ± 153							
	Shoot of *Cerdia congestiflora*			1175 ± 126							
	Shoot of *Brassica campestris*			1095 ± 84							
Zacatecas–Guadalupe Valley	Soil—Osiris	Microwave-digested samples. Analyzed using CVAAS ^(5)^			96.56						[76]
	Soil—La Zacatecana				47.95						
El Bote mine	Soil–slag	Hot-plate-digested samples. Analyzed using ICP-OES ^(1)^ and FAAS ^(2)^		1489.4			22.6	197.5	947.7	1197.1	[90]
	Brown slag			1015			7.7	184.4	116.5	665.8	
	Yellow slag			2061.9			30	12,313.1	1449	1499	
San Martin-Sombrerete mining district	Slag			1149			110	1534	4107	1397	
	Soil			702			93	769	1776	1893	
	Litter			856			80	1547	3616	866	
Fresnillo mining district	Soil–slag			695			44	186	827	2400	
	Slag			3388			73	344	2770	4189	
	Soil			16.3			3	15	283	599	
Noria de Ángeles mining district	Slag			665.9			20.8	114.1	668	1049.9	
	Soil			36.3			3.3	18	782	1032.3	
Guadalupe, Zoquite and San Jerónimo.	Agricultural soils	Microwave-digested samples. Analyzed using HGAAS ^(3)^ and CVAAS ^(5)^	3.3 to 182	10 to 868	0.05 to 198						[78]

^(1)^ ICP-OES = inductively coupled plasma atomic emission spectrometry; ^(2)^ FAAS = flame atomic absorption spectrophotometry; ^(3)^ HGAAS = hydride generation atomic absorption spectroscopy; ^(4)^ EDXRF = energy dispersive X-ray fluorescence; ^(5)^ CVAAS = cold vapor atomic absorption spectrophotometry.

## 4. Bioremediation Proposals at Sites Impacted by Mining Waste in Zacatecas

### 4.1. Study of Microbial Dynamics and Their Interaction with Soils or Solutions Contaminated with PTEs

Mining waste generates a new microenvironment characterized by low levels of organic carbon, low organic and inorganic nitrogen, high levels of metal cations and physicochemical conditions that limit microbial development [91]. However, there are studies related to the potential of microorganisms in the natural attenuation of sites contaminated with PTEs, in which the non-lethal selective pressure given by the environmental conditions that prevail in the sites is found to be a potential source of tolerant and/or resistant bacteria. This condition arises from microbial adaptation and evolution processes, guiding their use in bioremediation [92,93].

Some authors have evaluated the biostabilizing activity of microorganisms in historical mining wastes from Zacatecas; the formation of photosynthetic biofilm of microalgae in mining wastes from Concepcion del Oro mining district contaminated with Pb, Fe, Cu and Zn [94]; and the isolation of microorganisms from the genera *Staphylococcus hominis*, *Staphylococcus saprophyticus*, *Bacillus simplex*, *Bacillus* sp. and *Bacillus mojavensis* in Pb-contaminated soils from the La Zacatecana lagoon [95]. Both studies evaluated the modification of PTE speciation, establishing that microalgae favor the formation of complexes with carbonates and organic matter/sulfur, which is promoted by the destabilization of Fe oxides and the transition of PTEs to stable phases that limit their mobilization [94]. While the microorganisms isolated by Rodríguez-Sánchez et al. [95] carry out a biosorption/bioprecipitation process of Pb through their interaction with the functional groups of proteins and phosphate groups on the cell surface, inorganic compounds, such as pyromorphites and hydroxyapatite, were identified in the lyophilized biomass of these microorganisms.

### 4.2. Phytoremediation Studies in the State of Zacatecas

Mining residues lack an adequate physical structure, have weak water retention capacity and low nutrient content and contain PTEs; hence, they are extreme environments for plants [5,80]. Soil phytoremediation is a plant-based technology that aims to volatilize, stabilize, degrade, extract or inactivate soil contaminants. Native plants in areas impacted by the presence of mining wastes can accumulate PTEs present in the soil or waste (phytoextraction) and/or favor their immobilization in the rhizosphere, reducing their bioavailability without removing them from the site (phytostabilization) [96]. Metallophytes are a group of plants that grow in mineralized areas and have developed mechanisms that allow them to resist concentrations of metals that are toxic to most other plants. Some metallophytes are called hyperaccumulator plants because they can accumulate concentrations in their aerial tissues greater than 10,000 mg/kg of PTEs [5,96]. It is possible to use non-accumulating native plants that have adapted to grow in contaminated sites to revegetate degraded soils, establishing sustainable vegetation for natural attenuation by immobilizing PTEs. However, phytoremediation should be considered a slow biological process, because plants that survive these stress conditions have reduced growth and limited biomass production [97,98].

In the mining areas of Zacatecas, such as the community of Francisco I. Madero and the Fresnillo and Sombrerete mining districts, the presence of endemic plants with the capacity to tolerate Pb and As has been evaluated in soils with concentrations of up to 2898 ± 195 mg/kg of Pb and 2004 mg/kg of As (Table 5). In Francisco I. Madero, maximum Pb values were detected in the aerial parts of endemic plants such as *Amaranthus hybridus* (Quelite) at 2208 ± 136 mg/kg, *Buddleja scordioides* (Escobillon) at 1378 ± 153, *Cerdia congestiflora* at 1175 ± 126 and *Brassica campestris* (Mustasilla) at 1095 ± 84 mg/kg [5]. In the Fresnillo mining district ecological park, it has been shown that *Bouteloua gracilis* (Blue grama) has attributes for As phytoextraction processes, with concentrations in shoots of 1050 mg/kg and seeds of up to 1049 mg/kg. Additionally, trees such as *Schinus molle* and *Fraxinus uhdei*, which had been reforested after 15 years, generated a layer of organic matter that has improved fertility and erosion control in the mining wastes present [87]. Meanwhile, in the Sombrerete mining district, wild plants have been evaluated with high As concentrations in their shoots: 447 mg/kg for *Asphodelus fistulosus*, 342 mg/kg for *Pennisetum villosum* and 268 mg/kg for *Purshia mexicana*, with the latter reported as an As accumulator in the semi-arid region of Zacatecas [84].

On the other hand, in the Noria de Ángeles mining district (Figure 1a), native plants, such as *Acacia farnesiana*, *Prosopis laevigata*, *Schinus molle* and *Larrea tridentata*, have been evaluated and have been found to be tolerant to extreme temperatures, water scarcity and soils with concentrations of up to 95, 420 and 250 mg/kg of Cu, Pb and Zn, respectively (Table 5). Phytoextraction is observed by *Schinus molle*, with up to 68.4% of Cu, 4% of Pb and ~100% of Zn extracted [86]. Likewise, Martínez-González et al. [85] evaluated the phytoextraction of As, Cu, Pb and Zn using *Helianthus annuus* (sunflower) in Noria de Ángeles mining wastes with concentrations above the MPL established for industrial land use (Table 5). Average removal rates of 15% for As, 23% for Cu, 24% for Pb and 25% for Zn were observed after 180 days.

One of the areas of opportunity that has not been evaluated in historic mining areas of the state of Zacatecas is the sequestration of PTEs in phytogenic silica, commonly called phytoliths, which are mainly composed of SiO_2_·nH_2_O. This process involves complexation and/or co-precipitation of PTEs with monomeric silicic acid (H_4_SiO_4_) in different parts of the plants, in turn helping to stimulate antioxidant systems and improving efficiency in photosynthesis [99]. Silicon has been shown to mitigate the harmful effects of toxic elements like Cd or Zn on crop growth [100]. The efficiency of this process depends on a constant supply of plant available silicon (i.e., monomeric silicic acid), which ultimately originates from quartz (SiO_2_) weathering.

### 4.3. Interaction Between Microorganisms and Plant Mycorrhizae with Bioremediation Potential

Interactions between microorganisms and plant mycorrhizae play an important role in PTE biostabilization processes, as immobilization processes through chelation or methylation, dissolution, precipitation or absorption of PTEs take place in the rhizosphere [96]. In this regard, arbuscular mycorrhizae are ubiquitous symbiotic associations formed between arbuscular mycorrhizal fungi (AMF) and more than 90% of higher plants studied in terrestrial ecosystems [101], and are widely distributed in diverse environments, including degraded and polluted ecosystems [102]. These AMF facilitate the acquisition of mineral nutrients by host plants and contribute to the maintenance of plant community stability, productivity and ecosystem functioning. In addition, these AMF release various environmental stresses such as PTE toxicity, drought, salinity and soil compaction, allowing some plants to grow under stressful conditions and promoting natural soil restoration [103].

In Mexico, the establishment of plants and the presence of AMF and other rhizospheric fungi were studied in mine wastes from Zimapan, Hidalgo state, Mexico, using a holistic approach. It was determined that the genera *Glomus* and *Acaulospora* were the most abundant AMF found in the rhizosphere of plants from the families *Acanthaceae*, *Agavaceae*, *Amaranthaceae*, *Asteraceae* and *Brassicaceae*, used for the reforestation of these residues [104]. Likewise, in Temascaltepec, Mexico, a slag heap contaminated with high available Cd was studied, evaluating the morphological biodiversity of AMF spores and their participation in the stabilization of Cd through the use of plants from the *Fabaceae*, *Asteraceae* and *Poaceae* families. In this work, it is concluded that natural attenuation processes by plants, AMF (*Glomus*, *Gigaspora*, *Scutellospora* and *Acaulospora*) and mesofauna occur in this slag heap, modifying some important properties such as organic matter, color, pH and structural development, limiting the mobility of Cd [105].

In Zacatecas, these interactions have been evaluated in the rhizosphere of pioneer plants, such as *Bahia xylopoda* and *Viguiera linearis*, that can grow in the presence of high concentrations of heavy metals (11 mg/kg of Cd, 84.7 mg/kg of Pb, 35.2 mg/kg of Cu and 147 mg/kg of Zn), where the most abundant groups of microorganisms are members of the *Acidobacteria* and *Beta-proteobacteria* phyla, which are related to chemolithotrophic bacteria and sulfur-oxidizing microorganisms. These organisms use electron terminal acceptors from the mining waste, characteristic of an environment with high concentrations of secondary sulfate phases and lacking carbon and organic energy sources [106]. A similar role is played by nitrogen-fixing bacteria, such as *Paenibacillus*, that functionally exhibit high acetylene reduction activities but are sensitive to the presence of PTEs (Cr and Cu). However, some genera, such as *Paenibacillus graminis* BR_35 and *Paenibacillus borealis* BR_32, maintain significant acetylene reduction activity in the presence of high concentrations of Ni and Zn when associated with the rhizospheric zone of *Haplopappus* sp. and *Viguiera linearis* [107].

Some authors have also evaluated the effect of endophytic fungi on phytostabilization of Pb and Zn by *C. selloana* and Cd by *L. campestris*, *T. lunulata* and *A. fistulosus*. These plants grow on polymetallic wastes from the mining areas of Francisco I. Madero, El Bote mine and Vetagrande mining district (Figure 1b). Both arbuscular mycorrhizal fungi and dark septate endophytes promote the tolerance of these plants to stress conditions caused by the presence of PTEs [83].

Finally, González-Valdez et al. [88] tested seed germination and dry mass accumulation of five plant species (*Brassica napus L.*, *Brassica rapa L.*, *Celosia cristata L.*, *Tagetes erecta L.* and *Calendula officinalis L*.) cultivated on five mining wastes collected in Noria de Angeles and Vetagrande mining districts with high concentrations of As, Pb, Hg and Au (Table 5). These residues had high levels of Na, SO_4_^2−^ and electrical conductivity, chemical properties that impair seed germination and dry mass accumulation. Even in the presence of mining residues, *B. napus* showed high seed germination, tolerance, growth and total dry mass accumulation. Subsequently, González-Valdez et al. [108] evaluated the ability of *B. napus* to phytoextract Au, Ag and Cu from El Bote mine tailings, coupled with the application of NH_4_SCN or (NH_4_)_2_S_2_O_3_, and the combined inoculation of *Aspergillus niger* strains. The potential of *Aspergillus* strains to grow directly on the residues without affecting the growth of *B. napus* was made evident. The results indicate that the combined use of NH_4_SCN or (NH_4_)_2_S_2_O_3_ on *B. napus* inoculated with *Aspergillus* has positive effects during induced phytoextraction of Au, Ag or Cu.

### 4.4. Studies Associated with the Use of Organic Amendments

As the addition of soil with high organic matter and nutrient content is not economical for the revegetation of mining wastes, organic amendments are usually used as a substitute, as they help decrease the bioavailability of metals, provide a slow-release fertilizer that serves as a microbial inoculum, improve soil structure, reduce erosion and increase infiltration [109,110]. In addition, certain organic soil amendments, such as biochar, compost and animal wastes, have been recognized as PTE-immobilizing agents because of their abilities to restrain PTEs through different mechanisms [111,112]. An example of this process is biochar-assisted phytoremediation for the remediation of mine waste, tailings and PTE-contaminated soils. Biochar is a low-cost carbonaceous material that has extraordinary efficacy and applicability due to its porous structure and provides favorable conditions for fertility, water-holding capacity, pH, nutrients, carbon sequestration, microbial activities and pollution remediation [113,114]. The main mechanisms for removing PTEs using biochar as an organic amendment are as follows: (1) electrostatic interactions involving surface negative charge of biochar; (2) ion exchange due to the high cation exchange capacity of biochar; (3) complexation, where the biochar surface is rich in oxygen-containing functional groups; and (4) precipitation, a mechanism by which minerals present on the biochar surface precipitate with PTEs [114].

In Mexico, this process has been evaluated in mining waste for the remediation of Cu, Zn, Cd and Pb using biochar based on maize (*Zea Mays*) and *Jatropha curcas* as a phytoremediation plant, achieving an increase in the concentration of available P and water-holding capacity, positive changes to total nitrogen and organic matter content and decreased metal availability in the mine residue after 6 weeks of incubation [115].

In Zacatecas, Barajas-Aceves et al. [116] evaluated the bioremedial activity of *Brassica juncea*, *Acacia retinodes*, *Nicotiana glauca* and *Echinochloa polistaquia* in agricultural arid soils amended with Vetagrande mining district residues, bokashi, ethylenediaminetetraacetic acid (EDTA), compost and compost plus biosurfactants. The analyzed composite is an AMD generator and presented high concentrations of Pb, Zn, Cu, Cd and Cr (Table 5). The addition of mining residues to the soil inhibited CO_2_ production by 80%, dehydrogenase activity by 18% and nitrogen mineralization by 7%. The addition of bokashi reduced the inhibition of CO_2_ production and added Na and Mg to the soil, while the addition of EDTA increased CO_2_ inhibition and stimulated dehydrogenase activity and N mineralization. When bokashi was used, Pb presented a higher affinity for organic matter and inorganic colloids, which promote the stabilization of Pb through precipitation as hydroxides and oxides. *Acacia retinodes* and *Nicotiana glauca* grown on compost from mine wastes showed increases in dry biomass. High Pb concentrations in roots and the low translocation rate in *N. glauca* and *A. retinodes* indicate that they are suitable for phytostabilizing Pb and Zn.

## 5. Conclusions

The location, identification and characterization of historical mining residues in the state have been essential for understanding their mobilization processes, degree of dispersion and impacts on biota through their entry into trophic chains. However, a considerable number of historical mining wastes have not yet been characterized.

The historical mining districts most affected by the presence of PTEs in the state of Zacatecas are the Vetagrande, Zacatecas, Fresnillo and Concepcion del Oro mining districts; the alluvial zone of the Francisco I. Madero mine; and La Zacatecana lagoon. These areas present vestiges of the processing of metallic sulfide minerals, which have promoted their oxidation, and which leach, by the presence of AMD and the dispersion of PTEs, into agricultural soils, river sediments, surface water and groundwater. The presence of PTEs in arid and semi-arid areas is a latent problem that must be evaluated in depth; this implies the need for the constant monitoring of the quality of soils and of the surface and groundwater bodies that serve as sources of water for drinking or for agricultural and livestock use.

Phytoremediation studies that have been conducted in the state indicate that plants such as Schinus molle, Brassica napus, Brassica rapa, Celosia cristata, Tagetes erecta, Calendula officinalis, Cortaderia selloana and Lupinus campestris are ideal candidates in sites contaminated with Pb, As, Zn and Cd. These phytoremediation processes can be enhanced through the assisted use of microorganisms, organic amendments or low-toxicity chemical reagents that promote the phytostabilization of PTEs.

It is important to promote remediation strategies to limit the mobility of PTEs in the areas discussed in this work in order to safeguard the integrity of the environmental receptors near mining wastes in the state of Zacatecas.

## Figures and Tables

**Table 1:** Mining zones in Zacatecas classified by their mineralization type and deposit [3].

Mining District	Mineralization	Deposit	Mining Areas
San Julián	Au, Ag, Pb, Zn, Cu	Fissure filling	San Julián
Concepción del Oro	Au, Ag, Pb, Zn, Cu	Veins, mantles, breccias, chimneys, replacement and disseminated bodies	Peñasquito, Melchor Ocampo, Noche Buena y El Salvador
Miguel Auza–Juan Aldama	Ag, Au, Pb, Cu, Sn	Veins	Miguel Auza—Juan Aldama
Camino rojo–Nuevo Mercurio	Au, Hg	Fissure-disseminated fillings	Nuevo Mercurio
Francisco R. Murguía	Au, Ag, Pb, Zn, Sb	Irregular veins	Santa Rita, San Gregorio, Concordia, El Rosario y Nieves
Sombrerete–Chalchihuites	Au, Ag, Pb, Zn, Cu, Sn, Hg	Replacement bodies veins, chimneys and mantles	Sombrerete, San Martín, Chalchihuites
Saín Alto	Hg, Sn	Veins, lenses, irregular bodies and stockworks	Cerro Colorado, Bonancita, Sauz y Nuevo Mercurio
Villa de Cos	Mn, Hg, Sb, F, Ónix, Salt	-	La Abundancia, Manganita, La Prieta, San Felipe, Tenango, El Capirote y Sarteneja
Jiménez del Teúl	Au, Ag, Pb, Zn, Cu	Veins	Jiménez del Teúl
Fresnillo	Ag, Au, Pb, Zn	Disseminated bodies, chimney mantles and veins	Fresnillo
Valparaíso	Au, Ag, Sn, Bi	Veins	Valparaíso
Zacatecas	Ag, Pb, Zn, Cu, Cd	Veins and stratiform bodies	Zacatecas
Villanueva–Jalpa	Fluorite, Ag, Pb, Zn, Cu	Veins	Villanueva, Jalpa
Pánfilo Natera–Ojocaliente	Ag, Pb, Zn, Wollastonite	Veins and replacement bodies	Pánfilo Natera, Ojocaliente, Luis Moya
Noria de Ángeles	Pb, Ag, Zn	Irregular body	Noria de Ángeles-Real de Ángeles
Pinos	Au, Ag, Sn, Kaolín	Breccias and veins	Pinos
Mezquital del Oro	Au, Ag, Mn, Opal	Lenticular veins	Mezquital del Oro

**Table 2:** PTE concentration in soils (mg/kg), stream sediments (mg/kg) and water (ug/L) at different sites affected by mining waste around the world.

Country	Types of Mines or Mine Dump	PTEs							Ref
		As	Pb	Cr	Cd	Cu	Zn	Hg	
Soils									
United States	Arizona, mined tailings		2200		7.1	127	2000		[22]
United States	Nevada, Hg mine							170	[23]
South Africa	Krugersdorp, gold mine dump	1401	72.4	249	8.4		5340		[24]
Korea	AMD-contaminated soil		32.9	35.8	1.1				[25]
China	Copper mine dump	43.25	102.35	90.51	1.46	355.7	260.9		[26]
China	Lead–zinc mine		1093.03	30.91	7.14	57.8	867.1		[27]
China	Tongguan mine, gold mine dump	16	252		2.45	46.4	286	2.9	[28]
France	Les Malines mining district	338	34,289		225		30,364		[29]
Spain	Cartagena mining district, mine dumps	348	5950	70	67.2	323	23,361		[30]
Spain	Touro mine, Galicia, copper mine dump		19.3	118		911	78.2		[31]
Spain	Pb/Zn mining wastes		6761		43.7		32,287		[32]
Cyprus	Limni mine, copper mine dump		28.6		6.4	1534	4132		[33]
Tunisia	Jebel Ressas mining. Pb/Zn mining wastes		14,500		184	14.25	4240		[34]
Turkey	Gumuskoy, gold mine dump	4771	4320						[35]
Nigeria	The gold mining regions (Osun)		6.1	65.7	0.36	3.8	10.8		[36]
Algeria	Tamesguida, copper mine dump.	127.07	70.04	93.05		599.6	390		[37]
Bangladesh	AMD-contaminated soils	17.5	433						[38]
Oman	Gold mine dump		97	486	6	3240			[39]
Zambia	Copper mine dump		41.6			8980	83.3		[40]
India	Open cast mine-impacted soil		27.3	98					[41]
Portugal	Waste-impacted soil	38.5	31	93					[42]
Stream sediment									
Spain	Pb/Zn mining wastes		4650		31.5		12,772		[43]
Australia	Pb/Zn mining wastes		1796		8.7		6818		[44]
Mexico	Zimapan—Pb/Zn mining wastes		4785		193		1228		[45]
Mexico	Gold mine dump	1500	3908		66	90	8305		[46]
Water									
United States	Gold mine dump, groundwater	2700							[47]
Oman	Gold mine dump, surface water		1560	105	790				[39]

**Table 3:** Estimated amount of solid waste volume generated in the Zacatecas mining district (1982–2014) [51,52].

Mineral	Tons Extracted	Average Grades (g/tons)	Tons of Waste Generated
Gold	127.1	0.25	508,520,000
Silver	39,592.6	29	1,365,262,862
Copper	748,426	52	14,392,807,692
Lead	1,918,079	3.2	599,399,678
Zinc	4602.9	6.9	667,080,724

**Table 4:** PTE concentrations determined in biological samples, samples of drinking water and irrigation water in the mining areas of the state of Zacatecas.

Location	Type of Sample	Sample Processing and Analytical Technique	Concentration (µg/L)				Ref.
			As	Pb	Hg	F^−^	
Jerez	Tap water in primary schools	Microwave-digested samples. Determination by ICP-OES ^(1)^ coupled to a hydride generator	19 (8–62)			1800 (1600–2300)	[61]
El Visitador			22 (18–25)			1300 (800–2400)	
Guadalupe			78 (21–233)			450 (300–500)	
Ojocaliente			186 (125–298)			700 (600–800)	
Villanueva			6 (4–74)			400 (300–600)	
Tabasco			14 (8–25)			1900 (800–3000)	
Huanusco			26 (25–26)			1100 (400–1900)	
Calera aquifer (between Zacatecas and Fresnillo)	Water for human consumption	As was analyzed using FAAS, ^(2)^ F^−^ was determined by colorimetry method	18.5			1160	[62]
Morelos–Maderos mine	Agricultural irrigation water	Samples filtered, acidified and measured using FAAS ^(2)^ and HGAAS ^(3)^	158 ± 49	353 ± 21	2.7 ± 4		[63]
Fresnillo mining district	Water from stream beds and storage tanks	Microwave-digested samples. Analyzed using FAAS ^(2)^ and HGAAS ^(3)^	0.6 to 380	0.2 to 84	0.002 to 53		[64]
Calera aquifer flow system	Underground water	Samples filtered, acidified and measured using FAAS ^(2)^ and HGAAS ^(3)^	0.1 to 241.3			280 to 5400	[65]
Guadalupe–Bañuelos aquifer	Storm drain in Zacatecas	Samples filtered, acidified and measured using ICP-MS ^(4)^	27.2			730	[66]
	Well in the community of San Ramon		30			3200	
San Ramon aquifer system, Guadalupe–Zacatecas	Well water	Not reported	292 to 407			3050 to 3090	[60]
	Tap water (Zacatecas)		34 to 40			1570 to 1630	
	Tap water (Guadalupe)		42 to 85			1450 to 1720	
Vetagrande mining district	Blood in infants (MPL = 10 ug/dL)	Sample collected in capillary tube with EDTA. Sample measured using anodic stripping voltammetry		~13.6 ± 7 ug/dL			[67]

^(1)^ ICP-OES = inductively coupled plasma atomic emission spectrometry; ^(2)^ FAAS = flame atomic absorption spectrophotometry; ^(3)^ HGAAS = hydride generation atomic absorption spectroscopy; ^(4)^ ICP-MS = inductively coupled plasma mass spectrometry.

## Data Availability

All supporting data have been included in this study and are available from the corresponding authors upon request.
